# The impact of the experience of childhood poverty on adult health-risk behaviors in Japan: a mediation analysis

**DOI:** 10.1186/s12939-015-0278-4

**Published:** 2015-12-09

**Authors:** Maki Umeda, Takashi Oshio, Mayu Fujii

**Affiliations:** College of Nursing, St. Luke’s International University, 3-8-5 Tsukiji, Chuo-ku, Tokyo 104-0045 Japan; Institute of Economic Research, Hitotsubashi University, 2-1 Naka, Kunitachi, Tokyo 186-8603 Japan; Department of Education, Hokkaido University of Education-Hakodate, 1-2 Hachimanchou, Hakodate, Hokkaido 040-8567 Japan

**Keywords:** Childhood poverty, Inequalities in Health, Health behaviors, Socioeconomic status, Mediation analysis

## Abstract

**Background:**

The experience of childhood poverty has a long-lasting, adverse impact on physical health outcomes in adulthood. We examined the mediating effects of adult socioeconomic status (SES) and social support on the association between childhood poverty and adult health-risk behaviors.

**Methods:**

Cross-sectional data collected from Japanese community residents (*N* = 3836) were used. A binary indicator of the experience of childhood poverty was constructed by utilizing retrospectively assessed standard of living at age 15 and a set of parental SES variables. The associations of childhood poverty with smoking, lack of exercise, poor dietary habits, and excessive drinking at the time of survey were examined by logistic regression analysis. A mediation analysis was conducted to estimate the magnitudes of the mediating effects of adult SES and social support on these associations.

**Results:**

Adult SES and social support together mediated 64.0, 29.4 and 30.6 % of the impacts of the experience of childhood poverty on smoking, lack of exercise, and poor dietary habits, respectively. Educational attainment had the largest mediating effect (58.2 %) on the impact of the experience of childhood poverty on smoking.

**Conclusions:**

The results suggest that interventions and policies for supporting children living in poverty should aim to enhance their future SES and provide better social support, as this might improve their overall health.

## Background

Low socioeconomic status (SES) in childhood has a long-lasting adverse impact on numerous physical and mental health outcomes in adulthood [1–3]. Studies have shown that the experience of having low SES in childhood is associated with poor health in adulthood, largely due to harmful habitual behaviors, such as smoking, maintaining a sedentary lifestyle, poor dietary habits, and excessive drinking [4]. Childhood poverty is likely to reflect a various aspects of low SES in childhood and affect later-developing health-risk behaviors. Indeed, childhood poverty is often accompanied by parental absence or less parental structure (lack of rules or routines, such as regular bedtimes), poor quality housing, poor diet, and family conflicts. Poverty experienced in childhood is also correlated with an increased risk of academic underachievement and lower income in the future [5]. With the accumulation of these adversities, children may have reduced resources and opportunities to engage in healthy behaviors throughout their life course [6, 7].

Adult SES is one possible mediator of the impact of childhood poverty on adult health-risk behaviors. The experience of childhood poverty might reduce an individual’s likelihood of good educational attainment, occupational achievement, and future earning potential [5–7]. These factors could, in turn, lead to an increased likelihood of smoking, excessive alcohol consumption, poor dietary habits, and a sedentary lifestyle [8, 9]. A number of studies have examined the association of childhood SES, mostly measured by either the father’s occupation or education, with these health-risk behaviors in adulthood [10–13]. These studies generally found significant associations between lower childhood SES and health-risk behaviors, and discovered that these associations are largely explained by an individual’s own educational attainment and occupations. However, very few studies have specifically examined the impact of the experience of childhood poverty [14, 15], despite the fact that financial deprivation may have a distinctive effect on later health behaviors [16]. Furthermore, the mediating effect of adult SES on these associations has not been examined.

While social support is often considered to moderate the influence of childhood poverty on later health-risk behaviors, it could mediate this relationship [17, 18]. Childhood poverty may introduce individuals to certain social networks that could either promote or discourage positive health behaviors [19]. However, no previous study has examined the mediating effect of adult social support on the impact of the experience of childhood poverty on adult health-risk behaviors.

In the current study, we expand on previous research by examining a broader type of health-risk behavior using cross-sectional data collected from Japanese community residents. We particularly focused on smoking, lack of exercise, poor dietary habits, and excessive drinking, as these health-risk behaviors are considered key behavioral risk factors for a number of diseases and mortality [20]. To reduce recall bias and the subjectivity of retrospective self-assessment of childhood poverty, we constructed a binary variable of childhood poverty based on an ordered probit model. In this model, a categorical variable of self-reported childhood standard of living was estimated by the results obtained from multiple correspondence analysis of a set of parental SES measures. To further assess the relative importance of adult SES and social support as mediating factors, we used a mediation analysis wherein we examined the differences in the magnitude of the mediating effects among different sets of health-risk behaviors.

Childhood poverty is currently a major policy concern in Japanese society. The relative poverty rate (the ratio of individuals whose household-size-adjusted income is below the poverty line, i.e., 50 % of the median of the household-size-adjusted income of the population) of children in Japan was ranked 9th out of those of 35 developed countries, with 14.9 to 15.7 % of all children living in the country being under this poverty line [21, 22]. Single-parent families are at greater risk of experiencing poverty in Japanese society; in 2012, the relative poverty rate in single-parent households was 54.6 %, while that of the general Japanese population was 16.1 % [21]. A recent study also showed that Japanese children have relatively lower levels of material well-being (i.e., monetary and material deprivation) compared with children of other advanced countries, while other dimensions of child well-being, such as education and health-risk behaviors, are relatively favorable [22]. As such, empirical evidence is needed on the impact of childhood poverty on later life, as this will help support the development of mitigating policy measures.

## Methods

### Study sample

We conducted a secondary analysis on data from the Japanese Study of Stratification, Health, Income, and Neighborhood (J-SHINE) survey, the details of which are described by Takada et al. [23]. The J-SHINE survey was conducted between October 2010 and February 2011 in four municipalities in and around the Greater Tokyo Area. The municipalities were selected as examples of inner-sprawl urban and suburban regions, which were considered to suitably control for variations in structural and social environments. The J-SHINE targeted adults aged 25–50 years, as they are most likely to face a variety of interactions between sociodemographic/economic variables (e.g., marital status, childbearing, occupational status, and household income) and health. For each municipality, sixty sample units were selected proportionally to the registered population, and systematic sampling was conducted for each unit. Oversampling was performed among those aged in their 20s because of the expected lower response rate in this age stratum. We obtained official endorsement from each municipality, which helped us conduct the survey through their public relations activities. The total sample size was 4117 (response rate = 31.6 %). The questionnaire was computer-assisted and self-administered, unless participants requested a face-to-face interview. Participation in this study was voluntary, and written consent was obtained from each respondent. The Research Ethics Committee of the University of Tokyo Graduate School of Medicine approved the survey procedure (No. 3073-[1]).

### Measures

#### Childhood poverty: childhood standard of living and parental SES

We focused on retrospectively assessed participants’ recall of their standard of living at age 15 using a 5-point scale from 1 (*very poor*) to 5 (*very affluent*). Variables concerning parental SES included both parents’ educational attainment, presence in the home and occupational status, workplace size, and workplace position. Respondents were asked to report on these variables with reference to their living circumstances when they were 15 years old. This age was chosen because it marked the final stage of compulsory education and also it was, in most cases, the last point at which the children were fully dependent on their parents for financial support.

Parental educational attainment was grouped into the following categories: (1) junior high school, (2) high school, (3) college or above, and (4) unknown. Parental presence and occupational status included (1) regularly employed (including managers), (2) non-regularly employed, (3) self-employed, (4) unemployed, (5) homemaker, (6) unknown, and—in the case of parental absence—(7) deceased by the age of 15 years and (8) separated by that age. Workplace size was categorized based on the number of employees: (1) 1–9, (2) 10–99, (3) 100–999, (4) 1000 or more, (5) public sector, and (6) unknown. Parental position in the workplace included (1) staff (including self-employed), (2) senior staff, (3) chief, (4) manager, (5) director, (6) president/executive director, and (7) unknown. If parents were deceased or living separately from the respondent when he/she was 15 years old, we categorized parental workplace size and workplace position as “unknown” due to data limitations.

#### Health-risk behaviors

Four types of health-risk behaviors were measured: smoking, poor dietary habits, lack of exercise, and excessive drinking. Smoking was coded as positive when respondents reported being current smokers. Dietary habits were measured using 5 questions (“Do you eat breakfast every day,” “Do you try to eat vegetables,” “Do you try to cut down on sugar and salt intake,” “Do you try to purchase organic vegetables and additive-free food,” and “Do you try to eat nutritionally balanced meals?”) rated on a 5-point scale from 1 (*agree*) to 5 (*disagree*). We summed the scores of these 5 responses to determine a total score (range: 5–25; Cronbach’s α = 0.75) and defined poor dietary habits as a score of ≥16, which roughly corresponded with the lowest quartile of all responses.

Lack of exercise was measured by how often participants engaged in 10 min or more of physical activity, excluding incidental ones related to work, commuting, or other non-leisure behaviors, over the past year. The frequency categories and the percentage of participants who reported each category were as follows: every day (4.8 %), 5–6 days a week (4.9 %), 3–4 days a week (8.6 %), 1–2 days a week (20.5 %), once a month (19.4 %), or seldom (41.8 %). We defined a positive response to “seldom” as lack of exercise, considering that over 80 % of respondents did not meet the criteria for engaging in 10 min of exercise for more than 1–2 days per week, and 60 % reported not exercising more often than once a month. We also examined lack of exercise using two cut-off points: (1) once a month or less and (2) 1–2 days a week or less, the latter of which is closer to the nationally recommended standard of exercise (i.e., 30 min of exercise at least twice a week) [24].

Excessive drinking was assessed according to average daily alcohol intake calculated from the types and amounts of liquor consumed by respondents. Based on previous findings concerning the negative health effects of alcohol in Japan, an intake of 46 ml or more of pure alcohol per day was defined as excessive drinking [25].

#### Adult SES

Educational attainment comprised 4 categories: (1) junior high school completion, (2) high school graduation, (3) junior college (two-year college/vocational school) degree, and (4) college or higher educational degree. Occupational status included (1) regularly employed (including managers), (2) non-regularly employed, (3) self-employed, (4) unemployed, and (5) other (including homemakers).

Household income was assessed using 15 income bands. The median income for each band was equivalized by dividing the reported figure by the square root of the number of household members. This equivalized income was then divided into quintiles. For the respondents who did not report household income, we imputed it from estimated associations by regressing the respondent’s household income on their observed attributes (sex, age, marital status, educational attainment, occupational status, and the number of household members) and the surveyed municipality. The mean household income in the current survey was 6.85 million yen, including the 686 households whose income was missing and therefore imputed. This amount was slightly higher than 6.53 million yen, the mean household income in the Kanto Area (which includes the surveyed four municipalities) according to the government’s Family Income and Household Expenditure Survey in 2010 [26].

#### Adult social support

Assessment of social support was based on respondents’ perceived informational support. We asked the respondents, “How much helpful guidance do you receive from the following persons when you have problems or are in trouble?” The phrase, “the following persons,” referred to the individual’s (1) spouse/partner, (2) other co-residing family members, (3) non-co-residing family members or relatives, (4) neighbors, and (5) friends. The answers were scored on a 5-point scale from 1 (*a lot*) to 5 (*not applicable*). We reversed the order of responses and totaled the reversed scores for each source of support (Cronbach’s *α* = 0.95). Then, we categorized the score for each source of support into tertiles, indicating high, middle, and low social support.

#### Covariates

Data on the participants’ sexes, ages (25–29, 30–39, 40–50 years), marital status (having a spouse or partner), and surveyed municipalities (binary variables for each study site) were measured as covariates. We used ages as a categorical rather continuous variable to account for the possibility of their non-linear association with adult health-risk behaviors.

### Analytic strategy

#### Childhood poverty

We used a 5-point scale measure of standard of living at age 15, which the respondents retrospectively assessed. However, because this measure was subjective, it was not free from recall bias. To reduce the impact of recall bias, we constructed a variable of estimated childhood poverty based on parental SES variables, which were more objective and thus were expected to be less susceptible to recall bias.

This process involved five steps. First, we conducted multiple correspondence analysis [27] to explore the relationships among parental SES variables (i.e., parental presence/occupational status, educational attainment, workplace size, and workplace position). Second, we computed each respondent’s row coordinates based on the multiple correspondence analysis. Third, we estimated an ordered probit model to explain the subjective measure of standard of living in childhood by the row coordinates computed in the second step. This ordered probit model assumed that a continuous index representing a “true” level of childhood standard of living exists. Fourth, the continuous index of standards of living was linearly predicted by multiplying the independent variables and the estimated coefficients obtained in the third step. Finally, the lowest tertile of the estimated continuous index was assigned to each respondent as a binary variable of the experience of childhood poverty.

#### Mediation analysis

**Fig. 1:**
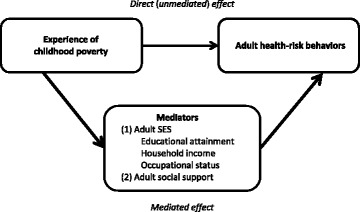
Conceptual framework of the mediation analysis

We conducted a mediation analysis [28, 29], the conceptual framework of which is illustrated in Fig. 1, to assess how much of the impact of the experience of childhood poverty on adult health-risk behaviors was mediated by adult SES and social support. The following variables were included in this analysis: (a) a binary variable of each health-risk behavior as a dependent variable; (b) a binary variable of the experience of childhood poverty as an independent variable; (c) binary variables of adult SES (i.e., educational attainment, household income, and occupational status) and social support as mediators; and (d) sex, age, marital status, and surveyed municipalities as covariates.

We followed the conventional steps of mediation analysis. First, we predicted each health-risk behavior by the experience of childhood poverty using logistic regression models (Models 1). The purpose of this step was to assess the total effect of the experience of childhood poverty on each health-risk behavior. Second, we estimated logistic regression models to predict each mediator by the experience of childhood poverty (Models 2). Finally, we estimated logistic regression models to predict each health-risk behavior by both childhood poverty and the mediators (Models 3). By comparing the results obtained for the second and third steps, we could clarify to what extent the effect of the experience of childhood poverty on health-risk factors was mediated by adult SES and social support independently. In each regression model, we included sex, age, marital status, and surveyed municipalities as covariates.

Furthermore, based on the results obtained from these models, we calculated the proportions of the direct (unmediated) effects of childhood poverty and the effects mediated by each mediator, along with their standard errors, using bootstrapping (with 5000 replications). This was to assess the relative importance and statistical significance of each mediator as well as the direct effect of childhood poverty on health-risk behaviors. To complete our statistical analysis, we used the Stata data analysis software (Version 13; StataCorp).

## Results

In brief, the results indicated that adult SES and social support together mediated a substantial portion of the impacts of the experience of childhood poverty on adult health-risk factors. Specifically, we found that the experience of childhood poverty was a reliable predictor of smoking, lack of exercise, and poor dietary habits (Table 3). Furthermore, educational attainment, household income, occupational status, and social support were associated with experience of childhood poverty (Table 4). After controlling for adult SES and social support, the strength of the association between the experience of childhood poverty and adult health-risk behaviors was reduced (Table 5). Adult SES and social support had the largest mediating effect on the impact of the experience of childhood poverty on smoking (Table 6). Among the mediators investigated, educational attainment had the largest mediating effect for smoking (Table 6).

We analyzed the data of 3836 respondents (men: *n* = 1777; women: *n* = 2059), excluding 281 responses that were missing data on key variables for the analysis. Table 1 displays the sociodemographic/economic characteristics of respondents and the proportions of respondents who reported health-risk behaviors in the total sample and in the subsamples according to experience of childhood poverty.

**Table 1:** Socio-demographic characteristics and health-risk behaviors of the total sample and subsample of childhood poverty

Socio-demographic characteristics		Proportion of childhood poverty	
Category	Subcategory	Total sample	Subsample of childhood poverty
Sex	Men	46.3	43.9
	Women	53.7	56.1
Age (years)	*M* (*SD*)	37.6 (7.22)	38.7 (6.9)
	25–29 years	18.4	14.1
	30–39	21.8	21.3
	40–50	20.9	24.1
Marital status	Married	70.8	73.6
	Unmarried/separated	29.2	26.4
Education	College or higher	43.7	28.4
	Junior college	12.3	11.3
	High school or lower	44.0	60.3
Occupation	Regular employment	51.6	48.8
	Non-regular employment	24.0	26.0
	Self-employed	5.7	6.3
	Unemployed	1.9	2.0
	Homemaker/other	16.8	16.9
Social support	High	31.4	32.1
	Middle	32.3	31.5
	Low	35.1	36.8
Smoking		23.7	27.2
Lack of exercise	Seldom	41.7	47.2
	Less than once per month	61.2	64.3
	Less than 1–2 days per week	81.2	81.6
Poor dietary habits		23.5	24.7
Excessive drinking		8.8	9.1
Number of observations		3836	1279

Table 2 shows the results of the multiple correspondence analysis. As shown in the table, the first dimension explained 41.8 % of the total inertia, while the explanatory power declined to 17.7 and 11.7 % for the second and third dimensions, respectively. These three dimensions captured 71.2 % of the variance of the set of variables denoting parental SES. We utilized the row coordinates corresponding to these three dimensions to explain participants’ recall of their standard of living at age 15, based on the ordered probit model. The rightmost two columns of Table 2 report the estimated coefficients—all of which were highly significant—on the row coordinates corresponding to the three dimensions and their standard errors. Based on these results, we constructed a binary variable of childhood poverty, as explained in the Methods section.

**Table 2:** Results of the multiple correspondence analysis for parental SES variables and ordered probit models to explain childhood poverty (*N* = 3836)

Multiple correspondence analysis for parental SES variables^a^				Ordered probit models to explain childhood poverty	
Dimension	Principal Inertia	Percentage	Cumulative percentage	Coefficient	(SE)
1	0.111	41.8	41.8	0.100^***^	(0.018)
2	0.047	17.7	59.5	0.248^***^	(0.018)
3	0.031	11.7	71.2	−0.218^***^	(0.018)

^a^ Total inertia was 0.265. Parental presence/occupational status, educational attainment, workplace size, and workplace position were used as parental SES

^***^ *p* < 0.001

Table 3 present the estimation results for Models 1, in which adult health-risk behavior was predicted by the experience of childhood poverty without controlling for the potential mediators (i.e., adult SES and social support). As the first step of the mediation analysis, we confirmed that the experience of childhood poverty was positively associated with smoking, lack of exercise, and poor dietary habits, but not with excessive drinking.

**Table 3:** Estimated associations between the experience of childhood poverty and adult health-risk behaviors (Models 1)^a^ (*N* = 3836)

		Smoking	Lack of exercise	Poor dietary habits	Excessive drinking
		OR (95 % CI)	OR (95 % CI)	OR (95 % CI)	OR (95 % CI)
Childhood poverty	Yes	1.53^*** ^(1.30–1.80)	1.55^*** ^(1.29–1.85)	1.48^*** ^(1.25–1.74)	1.12 (0.87–1.43)
	No	1	1	1	1

^a^All models were adjusted for sex, age, marital status, and surveyed municipalities

^***^ *p* < 0.001

At the second step of the mediation analysis, we estimated Models 2 to predict adult SES and social support by the experience of childhood poverty. For illustrative purposes, we re-categorized the adult SES and social support variables as binary variables and present their estimated associations with the experience of childhood poverty in Table 4. Specifically, we re-categorized adult SES and social support as follows: “high school or lower educational institution” and higher (educational attainment); “the lowest quintile of household income” and higher (household income); “non-regular employment or unemployment” and others (occupational status); and “the lowest tertile of social support total score” and higher (social support). We found that the experience of childhood poverty had positive bivariate associations with low levels of SES and social support in adulthood. Those who experienced childhood poverty were more likely to have a high school degree or less, earn within the lowest quintile of household income, stay in unstable employment (non-regularly employed or unemployed), and receive the lowest tertile of social support.

**Table 4:** Estimated associations between the experience of childhood poverty and adult SES and social support (Models 2)^a^ (*N* = 3836

Category		Adult SES			Adult social support
		Education	Household income	Occupational status	Lowest tertile
		High school or below	Lowest quintile	Non-regularly employed or unemployed	
		OR (95 % CI)	OR (95 % CI)	OR (95 % CI)	OR (95 % CI)
Childhood poverty	Yes	1.47^***^ (1.17–1.83)	1.58^***^ (1.33–1.88)	1.16 (0.99–1.37)	1.28^**^ (1.09–1.51)
	No	1	1	1	1

^a^All models were adjusted for sex, age, marital status, and surveyed municipalities

^***^ *p* < 0.001, ^**^ *p* < 0.01

After controlling for adult SES and social support in the third step of the mediation analysis, the strength of the association between the experience of childhood poverty and adult health-risk behaviors was reduced. By comparing the results in Tables 3 and 5, we found the odds ratios in response to the experience of childhood poverty declined from 1.53 to 1.21, 1.55 to 1.40, and 1.48 to 1.36 for smoking, lack of exercise, and poor dietary habits, respectively, after controlling for a set of mediators. We did not conduct this third-step analysis for excessive drinking, as it had no association with childhood poverty in the first step.

**Table 5:** Estimated associations between the experience of childhood poverty and adult health-risk behaviors controlling for adult SES and social support (Models 3)^a^ (*N* = 3841)

	Smoking	Lack of exercise	Poor dietary habits
	OR (95 % CI)	OR (95 % CI)	OR (95 % CI)
Childhood poverty	1.21^*^ (1.01–1.43)	1.40^***^ (1.22–1.62)	1.36^***^ (1.14–1.61)
Adult SES			
Educational attainment			
College or higher	1	1	1
Junior college	1.16 (0.82–1.64)	1.23 (0.98–1.54)	0.96 (0.69–1.32)
High school or lower	2.44^***^ (2.03–2.94)	1.14 (0.983–1.33)	1.25^*^ (1.04–1.49)
Household income			
1st quintile (highest)	1	1	1
2nd quintile	0.91 (0.70–1.18)	1.34^**^ (1.08–1.66)	1.11 (0.86–1.44)
3rd quintile	1.04 (0.79–1.36)	1.66^***^ (1.33–2.08)	1.15 (0.88–1.50)
4th quintile	1.21 (0.93–1.57)	1.59^***^ (1.27–1.98)	1.24 (0.95–1.61)
5th quintile (lowest)	1.13 (0.86–1.49)	1.88^***^ (1.49–2.37)	1.09 (0.83–1.42)
Occupational status			
Regularly employed	1	1	1
Non-regularly employed	1.10 (0.87–1.39)	0.98 (0.81–1.18)	0.97 (0.77–1.21)
Self-employed	0.74 (0.52–1.06)	0.83 (0.61–1.12)	1.05 (0.74–1.48)
Unemployed	0.58 (0.31–1.10)	0.84 (0.51–1.38)	1.02 (0.59–1.75)
Homemaker/other	0.60^**^ (0.44–0.83)	1.14 (0.92–1.42)	0.74^*^ (0.55–1.00)
Adult social support			
High	1	1	1
Middle	0.89 (0.72–1.10)	1.20^*^ (1.02–1.42)	1.28^*^ (1.04–1.58)
Low	1.12 (0.90–1.40)	1.54^***^ (1.29–1.85)	1.63^***^ (1.30–2.04)

^a^All models were adjusted for sex, age, marital status, and surveyed municipalities

^***^ *p* < 0.001, ^**^ *p* < 0.01, ^*^ *p* < 0.05

Finally, as shown in Table 6, the proportions of the impact mediated by adult SES and social support varied substantially across the health-risk behaviors: 64.0 % for smoking, 29.0 % for lack of exercise, and 30.6 % for poor dietary habits. Educational attainment mediated the largest proportion of the impact of the experience of childhood poverty on smoking (58.2 %) and poor dietary habits to a lesser extent (18.4 %), while household income mediated the largest proportion of the impact on lack of exercise (19.1 %). We found no significant mediating effect of occupational status for any of the health-risk behaviors, probably because of its close relationship with educational attainment and household income. The mediating effect of social support was modest, but statistically significant, for lack of exercise (7.1 %) and poor dietary habits (5.7 %), but was non-significant for smoking.

**Table 6:** Estimated propositions (%) of the direct and mediated effects of the experience of childhood poverty on adult health-risk behaviors^a^

	Smoking	Lack of exercise	Poor dietary habits
	% (95 % CI^b^)	% (95 % CI)	% (95 % CI)
Direct (unmediated) effect	36.0^c ^ (4.0–54.7)	70.6^c^ (53.9–83.3)	69.4^c^ (44.3–85.3)
Mediated effect via:			
Adult SES	62.6^c^ (44.2–94.1)	23.7^c^ (11.7–39.0)	23.5^c^ (8.5–45.2)
Educational attainment	58.2^c^ (40.7–90.2)	6.4 (−4.1–17.9)	18.4^c^ (4.8–37.5)
Household income	5.2 (−2.4–14.2)	19.1^c^ (11.6–31.0)	4.4 (−4.3–15.0)
Occupational status	−0.8 (−10.4–6.2)	1.8 (−8.2–1.8)	0.8 (−4.8–7.3)
Adult social support	1.4 (−1.6–5.3)	5.7^c^ (2.5–11.2)	7.1^c^ (2.9–14.9)
Total	64.0^c^ (45.3–96.0)	29.4^c^ (16.7–46.1)	30.6^c^ (14.7–55.7)
Total	100	100	100

^a^All models were adjusted for sex, age, marital status, and surveyed municipalities

^b^ Bias-corrected by bootstrap estimations with 5000 replications

^c^ indicates that the 95 % CI does not include zero

## Discussion

The current study highlighted the importance of childhood poverty in developing health-risk behaviors over time in a Japanese context. Our findings have provided empirical evidence that the experience of childhood poverty increases the likelihood of engaging in unhealthy behaviors and having an unhealthy lifestyle in adulthood [14–16]. The results were consistent with those of previous studies focusing on specific aspects of childhood SES, such as parental education and occupation, social class, and household income [4, 10–13]. Our findings were also supportive of the notion that unfavorable socioeconomic and psychosocial circumstances carried throughout the life course [5, 6] play an important role in the association between childhood SES and adult health behaviors [8, 9].

A key contribution of this study was the quantitative evaluation of each mediator of the impact of childhood poverty on adult health behaviors to determine its relative importance. This will help in prioritizing intervention targets. In our sample, adult SES and social support together mediated 29.4–64.0 % of the impact of childhood poverty on adult health-risk behaviors. We found that educational attainment was a key mediator, especially for smoking, which corroborates with existing literature [30]. This finding may be because children in poverty tend to be less successful in school, which could constrain the informational, financial, and social resources needed to acquire and maintain favorable health behaviors later in life [5]. We also found that social support had a modest, but significant, mediating effect on the impact of childhood poverty on poor dietary habits and lack of exercise. Social exclusion and adverse interpersonal experiences accompanying childhood poverty may hinder an individual’s access to social networks that provide positive support for healthy habits [17–19].

Our mediation analysis also showed that direct (unmediated) effects accounted for 36.0–70.6 % of the impact of the experience of childhood poverty of adult health-risk behaviors. These effects might be at least partially accounted for by mediators other than adult SES and social support. For instance, the psychological vulnerability and behavioral dysfunction caused by family turmoil, parental psychopathology, and dysfunctional coping styles often associated with poverty might also contribute to the increase in future health-risk behaviors [31].

In terms of analytic methodology, one novelty in the current study was the measurement of childhood poverty using limited longitudinal information in a cross-sectional study. Well-designed longitudinal studies have been conducted on the health impacts of childhood SES in New Zealand, the UK, the US, and Scandinavian countries [32, 33]. However, such research has not been conducted in most other countries, including Japan, because of limited resources for conducting long-term follow-up studies. The assessment of childhood financial condition in cross-sectional studies is often based on retrospective self-reported data, which is highly subjective and is vulnerable to recall bias [34]. In the current study, we constructed a variable of childhood poverty based on the results of a model wherein respondents’ retrospective and subjective assessment of the living standards in childhood were explained by parental SES variables that were more objective and thus likely less susceptible to recall bias [27].

It should be noted, however, that our measurement of childhood poverty had some limitations. First, it was not fully free from recall bias, because the parental SES variables were based on the respondents’ retrospective answers as well. Second, a binary variable of childhood poverty seems too limited to represent a concept that is inherently ordinal (i.e., related to distinct social classes), even though it had sufficient statistical power. Third, we measured standard of living only at age 15. As such, we may have failed to capture the potential diversity of changes in living conditions from infancy across respondents. Varied duration and timing of exposure to poverty may yield different consequences for health behaviors [35]. Thus, longitudinal data are needed to assess childhood poverty more rigorously and to overcome these limitations.

In addition to these limitations on the measurement of childhood poverty, the current study has several features that require us to be cautious in interpreting estimation results. First, our analysis was based on a cross-sectional dataset; thus, causality between adult health-risk behaviors and mediators is not assured. In other words, poor health status caused by health-risk behaviors might have affected adult SES and social support [36]. Second, the J-SHINE survey had a low response rate, and our respondents reported higher SES relative to the survey respondents of other studies. In addition, the reported health conditions tended to be poorer among the respondents excluded from our analysis due to missing data. These traits of the J-SHINE data may have resulted in an underestimation of the impact of low childhood and adult SES. Third, we defined “lack of exercise” as seldom exercising for 10 min or more, which is less frequent than the nationally recommended Japanese standard [24]. The results of this study indicate, however, that those who have experienced childhood poverty would be at a greater risk for exercising less than the national standard.

## Conclusions

Despite these limitations and drawbacks, the mediation analysis used in this study has added solid empirical evidence showing that improving educational attainment and household income as well as promoting social support may be an effective means of mitigating the negative health effects of childhood poverty. This study also extended previous research by demonstrating that health-risk behaviors of those who have experienced childhood poverty are mediated by adult SES and social support. Interventions and policies that support children living in poverty should work toward enhancing their future SES and providing better social support in order to improve their overall health. To further understand the impact of childhood poverty on adult health, future studies would benefit from the examination of other mediating factors, such as stressful life events experienced in young adulthood and individuals’ general psychological functioning [37].

## Abbreviations

SES: Socioeconomic status
J-SHINE: Japanese study of stratification, health, income and neighborhood
